# Intestinal Microbiota-Associated Metabolites: Crucial Factors in the Effectiveness of Herbal Medicines and Diet Therapies

**DOI:** 10.3389/fphys.2019.01343

**Published:** 2019-10-29

**Authors:** Yiliang Wang, Shurong Qin, Jiaoyan Jia, Lianzhou Huang, Feng Li, Fujun Jin, Zhe Ren, Yifei Wang

**Affiliations:** ^1^Guangzhou Jinan Biomedicine Research and Development Center, Institute of Biomedicine, College of Life Science and Technology, Jinan University, Guangzhou, China; ^2^Key Laboratory of Virology of Guangzhou, Jinan University, Guangzhou, China; ^3^Key Laboratory of Bioengineering Medicine of Guangdong Province, Jinan University, Guangzhou, China; ^4^College of Pharmacy, Jinan University, Guangzhou, China; ^5^Integrated Chinese and Western Medicine Postdoctoral Research Station, Jinan University, Guangzhou, China

**Keywords:** drug interventions, herbal medicines, traditional Chinese medicines, inter-individual differences, gut microbiota, metabolites

## Abstract

Although the efficacy of herbal medicines (HMs) and traditional Chinese medicines (TCMs) in human diseases has long been recognized, their development has been hindered in part by a lack of a comprehensive understanding of their mechanisms of action. Indeed, most of the compounds extracted from HMs can be metabolized into specific molecules by host microbiota and affect pharmacokinetics and toxicity. Moreover, HMs modulate the constitution of host intestinal microbiota to maintain a healthy gut ecology. Dietary interventions also show great efficacy in treating some refractory diseases, and the commensal microbiota potentially has significant implications for the high inter-individual differences observed in such responses. Herein, we mainly discuss the contribution of the intestinal microbiota to high inter-individual differences in response to HMs and TCMs, and especially the already known metabolites of the HMs produced by the intestinal microbiota. The contribution of commensal microbiota to the inter-individual differences in response to dietary therapy is also briefly discussed. This review highlights the significance of intestinal microbiota-associated metabolites to the efficiency of HMs and dietary interventions. Our review may help further identify the mechanisms leading to the inter-individual differences in the effectiveness of HM and dietary intervention from the perspective of their interactions with the intestinal microbiota.

## Background

The function of herbal medicines (HMs) and traditional Chinese medicines (TCMs) in the remedial and prophylactic management of human diseases has been recognized for a long time (Qiu, 2007; Fan et al., 2014; Wang et al., 2017; Xu et al., 2017; Nie et al., 2018; Wu and Tan, 2019), while the mechanisms of action of HMs remain largely unknown. Traditional studies focused on identifying the specific bioactive compounds in HMs, and such strategies have been successful in developing certain compounds isolated from HMs into novel drugs (Xu et al., 2017; Feng et al., 2019). However, most components extracted from HMs exhibit poor bioactivity and bioavailability (Xu et al., 2017; Feng et al., 2019). Indeed, the pharmacological activity of HMs largely depends on intestinal microbiota-dependent biotransformation (Xu et al., 2016; Aguilar-Toalá et al., 2018). Compared to the primary drugs, metabolites produced by the intestinal microbiota often exhibit greater pharmacological activity and are more easily absorbed (Inao et al., 2004; Hussain et al., 2016). Moreover, several components of HMs can serve as nutrition for the growth of specific microbiota and hence modulate the constitution of host intestinal microbiota (Xu et al., 2017; Feng et al., 2019). Therefore, the contribution of host intestinal microbiota-mediated biotransformation to the efficacy of HMs cannot be underestimated.

Indeed, the importance of the intestinal microbiota to human health and pathophysiology is indisputable. The beneficial effects of the intestinal microbiota are primarily contributed by the intrinsic constituents of the intestinal microbiota and the microbiota-associated metabolites, especially the subsets generated from beneficial bacteria (Rooks and Garrett, 2016; Bhat and Kapila, 2017; Hasegawa et al., 2017; Postler and Ghosh, 2017; Aguilar-Toalá et al., 2018; Cani, 2019; Silverman, 2019). The composition of the intestinal microbiota, and more specifically the metabolites generated through their biotransformation, has been shown to be closely associated with the large inter-individual differences observed in responses to drugs and dietary interventions (Coryell et al., 2018; Gong et al., 2018; Gopalakrishnan et al., 2018; Nie et al., 2018; Olson et al., 2018; Rothhammer et al., 2018; Routy et al., 2018; Maini Rekdal et al., 2019; Zimmermann et al., 2019a). Of note, *in vivo* drug activity, including pharmacokinetics and toxicity, is closely associated with the gut microbiota (Coryell et al., 2018; Gong et al., 2018; Gopalakrishnan et al., 2018; Nie et al., 2018; Olson et al., 2018; Routy et al., 2018; Maini Rekdal et al., 2019; Zimmermann et al., 2019a). Accumulating evidence reveals that intestinal microbiota are crucial contributors to the high inter-individual differences in dietary intervention efficacy in treating some refractory diseases (Flint et al., 2014; Thorburn et al., 2014; Buffington et al., 2016; Rioscovián et al., 2016; Hasegawa et al., 2017; Nie et al., 2018; Requena et al., 2018), such as the anti-seizure effect of the ketogenic diet (KD) (Olson et al., 2018). However, the interaction between HMs or diet therapy and the host intestinal microbiota remains largely unknown.

**FIGURE 1:**
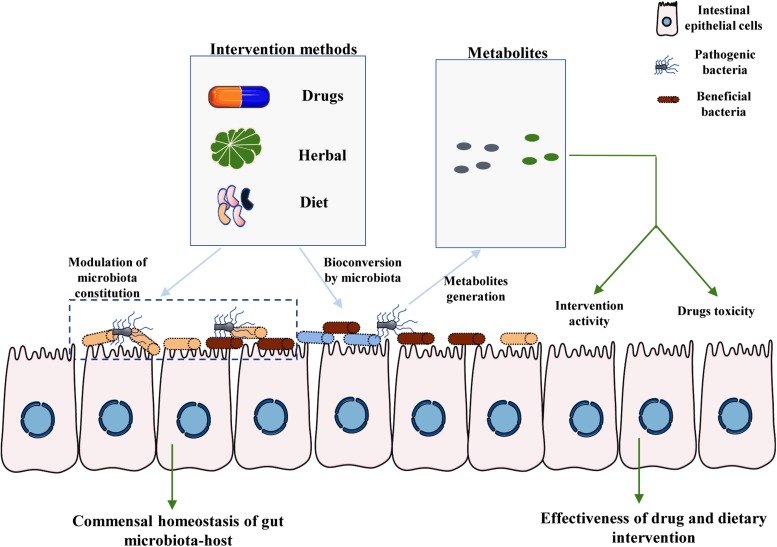
Crucial roles of intestinal microbiota-associated metabolites in the effectiveness of drug and dietary interventions. There are two main pathways by which the commensal microbiota affects the toxicity and efficacy of drug and dietary intervention. First, the specific components of HMs and diet provide nutrition to specific bacteria, including both beneficial bacteria and pathogenic bacteria, thereby modulating the homeostasis of the interaction between gut microbiota and the host. Moreover, particular components of the host diet and medicines can be metabolized by commensal microbiota to generate specific metabolites. The final metabolites may affect the toxicity and efficiency of drugs and dietary interventions, partly mediating the large inter-individual differences observed among hosts.

Owing to a range of factors, including host-intrinsic, host-extrinsic, and environmental factors, the taxonomic composition of the intestinal microbiota varies greatly among individuals (Tsb et al., 2018). It is critical to obtain a clear understanding of the links between HMs or dietary interventions and their metabolites from commensal microbiota. Herein, we mainly discuss the metabolites produced from TCMs and HMs by the intestinal microbiota (Figure 1). The contribution of commensal microbiota to the high inter-individual differences in dietary intervention efficacy is also briefly discussed. Our review further suggests that the effect of microbiota should be considered while developing new dietary guidelines or drugs for clinical application.

## Intestinal Microbiota-Associated Metabolites of the Compounds Isolated From HMs

Herbal medicines have significantly contributed to human health through disease prophylaxis and therapy (Xu et al., 2017; Feng et al., 2019). The term HM covers raw and processed plants such as the roots, leaves, flowers, berries, and/or seeds from one or more plants (Feng et al., 2019). Materials derived from animals, fungi, and minerals are also regarded as HMs in some traditions (Xu et al., 2017; Feng et al., 2019). Although most of the supposed pharmacological effects of HMs were determined by preclinical researches or even empirical study alone, multiple traditional medicine systems, such as TCMs, Ayurveda, and Islamic medicine, are dominated by HMs (Xu et al., 2017). However, the mechanisms of action of most HMs and the reasons for the different responses of different individuals remain unclear (Xu et al., 2016, 2017; Singh et al., 2017; Nie et al., 2018; Maini Rekdal et al., 2019). Of note, most of the chemicals derived from HMs exhibit poor bioactivity and bioavailability (Xu et al., 2017). However, intestinal microbes are involved in the metabolism of drugs (Maini Rekdal et al., 2019; Zimmermann et al., 2019a, b), especially the compounds extracted from HMs (Nie et al., 2018; Tong et al., 2018). Such biotransformation may contribute to explaining the great inter-individual differences in response to HMs because the constitution of gut microbiota varies among individuals (Xu et al., 2016; Tsb et al., 2018; Maini Rekdal et al., 2019). In this section, we mainly attempt to gain a more comprehensive and detailed understanding of the interactions between HMs and the intestinal microbiota. The role of microbiota in the *in vivo* activity and toxicity of chemical drugs is also discussed.

The compounds extracted from HMs that can be metabolized by the intestinal microbiota are mainly classified into subsets based on their chemical skeletons and include flavonoids, glycosides, terpenoids, anthraquinones, alkaloids, and organic acids (Table 1). Of these compounds, flavonoids are the most thoroughly studied, and most are degraded into flavone glycosides by the microbiota once the flavonoid enters the large intestine (Table 1). However, the final metabolites vary according to the specific medication and particular gut bacterial composition (Table 1). *Bifidobacteria* may be the group of microorganisms that can metabolize the widest range of compounds, including soy isoflavones, puerarin, ginsenoside, and sennoside (Table 1). Moreover, several specific bacteria can metabolize different compounds into the same metabolites. For instance, *Bifidobacterium* can metabolize both soy isoflavones and puerarin into daidzein (Table 1). Of note, the polyphenolics of berries and pomegranate fruit, a compound in unconventional HMs, can be metabolized by *Bifidobacterium pseudocatenulatum INIA p815* into urolithin A, which has multiple activities, including combating inflammation, oxidation, and aging, and enhancing gut barrier function (Singh et al., 2019). Collectively, the metabolism of HMs may not be highly dependent on a specific bacterium. However, the specific role of intestinal microbiota in the metabolism of HMs needs to be confirmed in clinical studies in the future, as the existing studies regarding their relationship refer only to preclinical studies.

**TABLE 1:** Metabolites produced by intestinal bacteria from HMs.

**Drugs**	**Bacteria involved (if available)**	**Metabolite**	**Function (determined by preclinical studies alone)**	**References**
**Flavonoids and their derivates**				
General flavonoids	*Clostridium* spp.	Desaminotyrosine	Modulation of type I interferon.	Schoefer et al., 2003; Ozdal et al., 2016; Steed et al., 2017
Soy isoflavones	*Bifidobacterium breve* 15700	Equol Daidzein	Modulation of platelet function. Prevention of thrombotic events.	Braune and Blaut, 2011; Elghali et al., 2012
Puerarin	*Bifidobacterium longum* BB536 *Lachnospiraceae* strain CG19-1	Daidzein		
Mangiferin	*Bacteroides* sp. MANG *Lachnospiraceae* strain CG19-1	Norathyriol	Suppresses skin cancers. Reverses obesity-induced and high-fat diet-induced insulin resistance.	Sanugul et al., 2005; Braune and Blaut, 2011; Li et al., 2012; Ding et al., 2014
Hesperidin	Uncertain	Hesperetin	Anti-inflammatory and antioxidation effect.	Yang et al., 2002, 2012; Lee et al., 2004; Alok et al., 2017
Kaempferitrin	Uncertain	Kaempferol 3-O-α-L-rhamnoside Kaempferol 7-O-α-L-rhamnoside Kaempferol p-Hydroxybenzoic acid	Anxiolytic activity.	Vissiennon et al., 2012
Baicalin	Uncertain	Baicalein Oroxylin A	Anti-pruritic Anti-inflammatory	Trinh et al., 2010; Myung-Ah et al., 2012; Serra et al., 2012
**Glycosides**				
Ginsenoside	*Bacteroides* sp. *Bifidobacterium* sp. *Fusobacterium* sp.	Rg3, Rh2, and compound K	Cardio-cerebral vascular system protection. Nervous system protection. Anti-tumor function.	Odani et al., 1983; Eunah et al., 2002; Bae et al., 2004; Li et al., 2010; Jung et al., 2012
Glycyrrhizin	Uncertain	Glycyrrhetic acid monoglucuronide	Anti-inflammatory, anti-ulcer, antiallergic, anti-dote, anti-oxidant, anti-tumor, and anti-viral activity.	Akao, 2000; Baltina, 2003
**Anthraquinone**				
Sennoside	*Lactic acid bacterial* strains *Bifidobacterium* strains	Rheinanthrone	Restrictive effect on diarrhea.	Matsumoto et al., 2012; Takayama et al., 2014
Barbaloin	*Eubacterium* strain Bar	Aloe emodin Anthrone	Restrictive effect on diarrhea.	Akao et al., 1996
Terpenoids				
Geniposide	Uncertain	Genipin	Protective effect on chemically induced liver injury.	Inao et al., 2004; Kang et al., 2012; Khanal et al., 2012; Jin et al., 2014
Paeoniflorin	Uncertain	Paeoniflorin, Paeoni lactone glycosides, Paeonimetabolin I, II, III	Protective effect on the cardiovascular system and nervous system.	Hsiu et al., 2003; Wozniak et al., 2007; Abdel-Hafez et al., 2010; Merenstein et al., 2010
Alkaloids				
aconitine	Uncertain	8-Butyryl-14-benzoylmesa-conine 8-Propionyl-14-benzoylaconine 8-Butyryl-14-benzoylaconine 8-Valeryl-14-benzoylmesaconine	Anti-inflammatory Painkillers	Borcsa et al., 2011; Xin et al., 2012
**Organic acids**				
Chlorogenic acid	*Escherichia coli Bifidobacterium lactis Lactobacillus gasseri*	Caffeic acid Quinic acid M-coumaric acid Ferulic acid Isoferulic acid Hippuric acid 3-Hydroxyhippuric acid	Antioxidant Anticarcinogenic Suppresses the adherence of pathogenic bacteria such as *H. pylori*.	Gonthier et al., 2003; Gotteland et al., 2006; Couteau et al., 2010; Rio et al., 2010; Ludwig et al., 2013; Tomas-Barberan et al., 2014
Dark tea	Uncertain	4-hydroxybenzoic acid 8-C N-ethyl-2-pyrrolidinone substituted flavan- 3-ols	Improvement of age-related neurodegenerative diseases Antioxidant capacity	Cai et al., 2018

In addition to the HMs, the gut microbiota is also closely associated with the *in vivo* activity of chemical drugs. Given that prior influential studies have revealed the gut microbes involved in drug metabolism and their potential genes (Zimmermann et al., 2019a, b), we briefly discuss the role of microbiota-mediated biotransformation in drug activity and toxicity through introducing several representative drugs (Table 2). For instance, gut microbes have been suggested to be crucial factors in the conversion of L-dopa to dopamine (Maini Rekdal et al., 2019). The bioconversion of L-dopa to dopamine depends on a pyridoxal phosphate-dependent tyrosine decarboxylase from *Enterococcus faecalis* followed by transformation of dopamine to m-tyramine by a molybdenum-dependent dehydroxylase from *Eggerthella lenta* (Maini Rekdal et al., 2019). In addition, the gut microbiota is responsible for varying responses to simvastatin treatment, resulting in vast differences in the hypolipidemic effect of simvastatin among patients (Krauss et al., 2013; He et al., 2017). Furthermore, although PD-1 inhibitors exhibit potent activity against cancer by blocking a “checkpoint” molecule on T cells, only 25% of patients respond well to PD-1 blockers. The gut microbiota is a crucial factor in determining the response of an individual to various treatments (Gopalakrishnan et al., 2018; Routy et al., 2018). Gut microbes are also a crucial factor affecting the *in vivo* drug toxicity. For example, diurnal variation in acute liver injury caused by acetaminophen is partly mediated by 1-phenyl-1,2-propanedione, a metabolite of acetaminophen generated by gut microbiota (Gong et al., 2018). Interestingly, acetaminophen hepatotoxicity can be reduced through postbiotic-induced autophagy by *Lactobacillus fermentum* (Dinic et al., 2017), which demonstrates that different bacteria play distinct roles in the toxicity of the same drug. These findings suggest that an understanding of the interaction between intestinal microbiota and drug metabolism is critical for developing new drugs that are efficacious, which is significant for the frequent emergence of drug-resistance.

**TABLE 2:** Metabolites produced by intestinal bacteria from chemical drugs.

**Drugs**	**Bacteria involved (if available)**	**Metabolite (if available)**	**References**
Acetaminophen	*Citrobacter freundii; Escherichia coli*	1-phenyl-1,2-propanedione	Gong et al., 2018
Tacrine	Bacteria with coding beta-glucuronidases	–	Bisanz et al., 2018; Yip et al., 2018
SN-38 glucuronide	Bacteria with coding beta-glucuronidases	SN-38	Wallace et al., 2010; Spanogiannopoulos et al., 2016; Guthrie et al., 2017; Bisanz et al., 2018
Sulfasalazine	*Bacteroides* sp., *Enterococcus faecalis* and two *Lactobacillus* sp.	5-aminosalicylic acid	Spanogiannopoulos et al., 2016
Prontosil	-	triaminobenzene and sulfanilamide	Fuller, 1937
Digoxin	*Eggerthella lenta* coding cardiac glycoside reductase (cgr) operon	dihydrodigoxin	Spanogiannopoulos et al., 2016
Non-steroidal anti-inflammatory drugs (including diclofenac, indomethacin, and ketoprofen)	Bacteria with coding beta-glucuronidases (such as *Proteobacteria*, *Firmicutes* and *Actinobacteria phyla*)	Aglycon etc.	Spanogiannopoulos et al., 2016
Melamine	*Klebsiella terrigena*	cyanuric acid	Xiaojiao et al., 2013; Spanogiannopoulos et al., 2016
L-dopa	*Enterococcus faecalis* pyridoxal phosphate-dependent tyrosine decarboxylase	dopamine	Maini Rekdal et al., 2019
Dopamine	*Eggerthella lenta* molybdenum-dependent dehydroxylase	m-tyramine	Maini Rekdal et al., 2019
Simvastatin	–	–	Krauss et al., 2013; He et al., 2017

## Gut Microbes: Crucial Factors for the Function of TCM

It has long been known that TCM is effective for treating many human diseases, including influenza virus infection, cancer, diabetes, and cardiovascular diseases (Qiu, 2007; Fan et al., 2014; Wang et al., 2017; Xu et al., 2017; Nie et al., 2018; Wu and Tan, 2019). The fundamental functions and applications of TCM depend on the compatible application of herbal formulas (FuFang in Chinese) based on ancient empirical philosophies such as *Yin-Yang* (Dong et al., 2018). However, the mechanisms of action of TCM remain largely unclear or unknown. Recent insights into TCM have focused on its interactions with the gut microbiota (Xu et al., 2017; Feng et al., 2019; Wu and Tan, 2019). Firstly, the carbohydrates in HMs cannot be digested by the human body, while the human gut microbiome encodes thousands of carbohydrate-active enzymes to digest herbal carbohydrates (Xu et al., 2017; Lu et al., 2019). Secondly, the non-carbohydrate bioactive compounds in TCM, particularly triterpene glycosides, flavonoids, isoflavones, iridoid glycosides, alkaloids, and tannins, have poor lipophilicity, high hydrogen-bonding capacity, and high molecular flexibility, which limit the bioavailability of TCM (Xu et al., 2017). However, these non-carbohydrate compounds can be metabolized into several metabolites by the gut microbiota, increasing the efficiency of intestinal absorption and thereby improving their bioavailability (Xu et al., 2017). Moreover, most TCM formulas can reshape the structure of commensal flora, such as by increasing the level of beneficial bacteria and reducing the abundance of harmful bacteria (Table 3). Of note, the enrichment of beneficial gut microbes and the reduction of harmful gut microbes is not merely a result of disease symptom improvement, because the recovery of the balance of the gut microbiota usually occurs before an improvement in the disease symptoms (Xu et al., 2015). Collectively, the efficacy of TCMs may be the comprehensive outcome of both reshaping the microbiota structure and the complex interaction between intestinal microbiota and multiple chemical substances in TCMs.

**TABLE 3:** Effect of Traditional Chinese medicines (TCM) formulas on the constitution of commensal microbiota and host metabolisms in indicated diseases.

**TCM formulas**	**Effect on gut microbiota**	**Effect on host metabolisms**	**Function**	**References**
Tiansi Liquid	Increase: *Ruminococcaceae, Lactococcus, Lactobacillus, Lachnospiraceae_NK4A136_group*	Increased the level of kynurenic acid and 5-HT	Improve hydrocortisone-induced depression	Cheng et al., 2018
Qushi Huayu Fang	Increase: *Collinsella*; Decrease: *Escherichia/Shigella* ratio	Increased the level of SCFAs	Improve non- alcoholic fatty liver disease	Yin et al., 2013
Bawei Xileisan	Increase: *Bacteroides* and *Lactobacillus*	–	Treatment of ulcerative colitis	Wen et al., 2016
Red Ginseng and Semen Coicis	Increase: *Bifidobacterium* and *Lactobacillus* (*in vitro*)	–	Relieve the symptoms of ulcerative colitis	Guo et al., 2015
Gegen Qinlian Decoction	Increase: *Faecalibacterium*, *Gemmiger*, *Bifidobacterium*, *Lachnospiraceae incertae sedis*, and *Escherichia*; Decrease: *Alistipes*, *Odoribacter, Parabacteroides, Bacteroides*, and *Pseudobutyrivibrio*		Treatment of T2D	Xu et al., 2015
ZiBuPiYin recipe	Increase: *Roseburia* and *Coprococcus*	–	Improve psychological-stress-induced diabetes-associated cognitive decline	Chen et al., 2014; Gu et al., 2017
Oil tea	Increase: *Lachnospiraceae*	Limited the elevation of postprandial blood glucose and lowered the levels of fasting blood glucose	Antidiabetic effects	Lin et al., 2018
Zengye decoction	Decrease: *Desulfovibrio*, *Ruminococcus*, *Prevotella* and *Dorea*; Increase: *Oxalobacter*, *Clostridium* and *Roseburia*	Inhibited methane metabolism, strengthened the physiological function of glutathione	Treatment of constipation	Liu et al., 2019
Moxibustion	Increase: *Bifidobacterium* and *Lactobacillus*; Decrease: *Escherichia coli* and *Bacteroides fragilis*;	–	Treatment of ulcerative colitis	Wang et al., 2012

The most typical example of this is the excellent efficacy of TCMs in the management of type 2 diabetes (T2D) (Xu et al., 2015; Nie et al., 2018; Tong et al., 2018; Cheng F. R. et al., 2019; Cheng J. et al., 2019; Han et al., 2019; Li et al., 2019; Lu et al., 2019; Shi et al., 2019; Wu et al., 2019; Yuan et al., 2019). The major component of HMs, such as the polysaccharides extracted from *Hirsutella sinensis*, provides nutrition to specific bacteria, thereby modulating the constitution of the intestinal microbiota to improve T2D (Xu et al., 2015, 2017; Nie et al., 2018; Tong et al., 2018; Wu et al., 2019; Table 3). Of note, a multicenter, randomized, open-label clinical trial revealed that metformin and the Chinese herbal formula AMC (including *Rhizoma Anemarrhenae*, *Momordica charantia*, *Coptis chinensis*, *Salvia miltiorrhiza*, red yeast rice, *Aloe vera*, *Schisandra chinensis*, and dried ginger) may ameliorate T2D with hyperlipidemia by enriching beneficial bacteria, including *Blautia* and *Faecalibacterium* spp. (Tong et al., 2018). In addition, treatment of Gegen Qinlian Decoction (GQD), another TCM formula, can enrich the gut in beneficial bacteria such as *Faecalibacterium* spp., which is associated with the anti-diabetic effect of GQD (Xu et al., 2015; Table 3). Indeed, under fermentation by the intestinal microbiota, HMs can be metabolized into various chemical substances with wide-ranging activities that improve host health (Yang et al., 2012; Nie et al., 2018; Wu et al., 2019) and jointly enhance the gut barrier, control insulin resistance, and reduce inflammation in the host (Nie et al., 2018). Furthermore, HMs regulate many complex chemical interactions in the gut, thereby maintaining a healthy gut ecology (Nie et al., 2018), which is important in recovery from gut dysbiosis. However, whether these altered microbiotas metabolized specific components in TCMs into functional molecules remains uncertain. Metabolomics analysis is an ideal method for determining the altered microbiota-associated metabolites of TCMs.

## Effect of Intestinal Microbiota-Associated Metabolites on the Efficiency of Dietary Therapy

Dietary interventions have become an effective method for treating some refractory diseases, with the effects being associated with the commensal microbiota of the host Richards J. L. et al., 2016; Wu et al., 2016). The KD has long been known to exhibit high efficacy against refractory seizure, despite the response rate being low among tested patients (Kwan and Brodie, 2000; Olson et al., 2018). A recent influential study revealed that the gut microbiota was responsible for the high inter-individual differences observed in the anti-seizure effect of the KD (Olson et al., 2018). Ketogenic diet-associated *Akkermansia* and *Parabacteroides* confer seizure protection to mice fed a controlled diet by reducing the level of gamma-glutamyl amino acids and increasing the GABA and glutamate content in the brain (Olson et al., 2018). In addition, a Mediterranean diet, which is based on the high consumption of cereals, fruit, vegetables, and legumes, has been associated with the prevention of cardiovascular diseases and asthma (Castro-Rodriguez et al., 2008; Estruch et al., 2013; Blanco Mejía et al., 2019). The Mediterranean diet increases the abundance of *Lactobacillus* in the mammary gland microbiota and subsequently elevates the levels of bile acid and bacterial-modified metabolites in breast cyst fluid (Shively et al., 2018). However, the beneficial effects of the Mediterranean diet on human health also depend, in part, on non-bacterial metabolites, especially ω-3 fatty acids, which exert larger anti-inflammatory effects (Thorburn et al., 2014). Further, given that the Mediterranean diet is rich in fiber, SCFAs may mediate the beneficial effect of this diet, since the administration of SCFAs is associated with significant improvements in cardiovascular diseases (Richards L. B. et al., 2016); this requires further research. Of note, in the gastrointestinal tract of human patients with type II diabetes, the administration of *Bifidobacterium* increases the abundance of *Akkermansia muciniphila*, with both microbes being able to generate SCFAs, thereby improving insulin resistance and limiting inflammation and consequently improving the symptoms of obesity (Cani, 2019). Furthermore, arsenic poisoning arising from the ingestion of contaminated food and drinking water is a challenging disease to treat (Coryell et al., 2018). A promising finding is that gut microbes, especially *Faecalibacterium*, provide full protection against acute arsenic toxicity in a mouse model (Coryell et al., 2018).

However, some of the observed dietary effects have not yet been associated with specific intestinal microbes or with specific metabolites. For instance, a maternal high-fat diet negatively impacts the social behavior of offspring, resulting in a deficiency in synaptic plasticity in the ventral tegmental area and in oxytocin production, but the administration of *Lactobacillus reuteri* restores oxytocin levels, synaptic plasticity, and healthy social behaviors in mice (Buffington et al., 2016). It has also been recognized that a Malawian diet may induce kwashiorkor, an enigmatic form of severe acute malnutrition. In a study involving 317 Malawian twin pairs, researchers found that an altered gut microbiota constitution in response to the Malawian diet significantly contributed to the development of kwashiorkor, although the mechanism involved remains unknown (Smith et al., 2013). Notably, oligosaccharides were less abundant in the milk from mothers of severely stunted infants, and the administration of sialylated milk oligosaccharides reversed infant undernutrition in a microbiota-dependent manner (Smith et al., 2013). Such results were also confirmed in piglets that received the same diet as the human infants (Charbonneau et al., 2016), suggesting that microbiota associated-metabolites of oligosaccharides may be a crucial factor in such processes. In young children, a negative association between dietary fiber and plasma insulin levels has been observed only in those whose gut microbiota showed a high abundance of *Bacteroides* and *Prevotella* and not in those whose gut microbiota exhibited a higher proportion of *Bifidobacterium* (Zhong et al., 2019). This suggests a potential function for *Bacteroides* and *Prevotella* in elevating insulin levels. Indeed, convincing epidemiological studies have indicated that specific dietary components may be crucial for the pathogenesis of some diseases such as asthma and allergies (Eder et al., 2006; Graham, 2006). For example, a carnitine-rich diet induces the symptoms of atherosclerosis in a gut microbiota-dependent manner in humans and mice (Koeth et al., 2013). Specifically, the gut microbiota in humans and mice mediates the metabolism of dietary choline and phosphatidylcholine to produce trimethylamine, which is further transformed into trimethylamine-*N*-oxide by hepatic flavin monooxygenases, thereby promoting the development of atherosclerosis. However, the specific microbial taxa contributing to this process require further investigation.

## Conclusion and Future Perspective

The beneficial effect of HMs and dietary therapy in several refractory diseases is generally appreciated, but the underlying mechanisms involved remain obscure. However, their interaction with the host microbiota seems to be a critical factor in such processes. Indeed, a growing number of studies indicate that the commensal microbiota plays a crucial role in maintaining host health and that the constitution of the intestinal microbiota exhibits large inter-individual differences. Moreover, most components in HMs and dietary interventions can modulate the constitution of the microbiota, which may disrupt or maintain homeostasis in the host. Collectively, it is not surprising that the gut microbiota, and especially microbiota-associated metabolites, may be a crucial mediator linking HMs or dietary therapy and the physiological status of the host. Therefore, it is important to consider the effects of biotransformation by commensal microbiota when designing herbal formula dietary therapy to achieve optimal success in treating diseases, particularly in the case of precision medicine. It is also essential to determine the optimal timing of administrating HMs and specific diets, in particular given that the composition of the gut microbiota exhibits diurnal variation. Indeed, microbiota-associated metabolites have several attractive properties, including known chemical structures and long shelf lives (Aguilar-Toalá et al., 2018). In particular, these metabolites are able to mimic the health effects mediated by probiotics while avoiding the administration of live bacteria, which can produce harmful reactions such as the local inflammatory response induced by the administration of *Salmonella* (Tsilingiri et al., 2012). However, the importance of postbiotics does not diminish the beneficial effect of probiotics when there is stable colonization of the gut, because live bacteria undoubtedly provide more metabolites than can be provided using postbiotics. The future of next-generation probiotics lies not only in supplementation using beneficial bacteria strains but also in providing and maintaining the ecological context necessary to sustain them. The direct administration of these probiotic-associated metabolites should provide a great advantage over traditional probiotics for several types of patients, including those harboring intestinal pathogens. Furthermore, since metabolites from the intestinal microbiota can also partially mediate the toxicity of some medicines *in vivo*, it will also be valuable to further examine these associations in order to assist in developing novel approaches to reducing the toxicity of HMs and TCMs.

## Author Contributions

YlW contributed to the conception, design, collection and assembly of references, discussion, interpretation, and writing of the manuscript. SQ contributed to the collection and assembly of references, interpretation of the article, and writing of the manuscript. JJ, LH, and FL contributed to the collection and assembly of references. FJ, ZR, and YfW contributed to the conception, design, interpretation of the article, and the final article approval.

## Conflict of Interest

The authors declare that the research was conducted in the absence of any commercial or financial relationships that could be construed as a potential conflict of interest.
